# Biomass gasification to syngas in thermal water vapor arc discharge plasma

**DOI:** 10.1007/s13399-023-03828-3

**Published:** 2023-02-13

**Authors:** Andrius Tamošiūnas, Dovilė Gimžauskaitė, Mindaugas Aikas, Rolandas Uscila, Vilma Snapkauskienė, Kęstutis Zakarauskas, Marius Praspaliauskas

**Affiliations:** 1 Plasma Processing Laboratory, Lithuanian Energy Institute, LT-44403 Kaunas, Lithuania; 2 Laboratory of Combustion Processes, Lithuanian Energy Institute, LT-44403 Kaunas, Lithuania; 3 Laboratory of Heat Equipment Research and Testing, Lithuanian Energy Institute, LT-44403 Kaunas, Lithuania

**Keywords:** Biomass, Wood pellets, Thermal arc plasma, Syngas, Waste-to-energy

## Abstract

This study investigated biomass (wood pellets) gasification to syngas using direct current (DC) thermal arc plasma at atmospheric pressure. Water vapor was used as a main gasifying agent and a plasma-forming gas. The biomass gasification system was quantified in terms of the producer gas composition, the tar content, the H_2_/CO ratio, the carbon conversion efficiency, the energy conversion efficiency and the specific energy requirements. It was found that the gasification performance efficiency was highest at the water vapor-to-biomass ratio of 0.97. The producer gas was mostly composed of H_2_ (43.86 vol.%) and CO (30.93 vol.%), giving the H_2_/CO ratio of 1.42 and the LHV of 10.23 MJ/Nm^3^. However, high content of tars of 13.81 g/Nm^3^ was obtained in the syngas. The yield of H_2_ and CO was 48.31% and 58.13%, respectively, with the highest producer gas yield of 2.42 Nm^3^/kg biomass. The carbon conversion efficiency and the energy conversion efficiency were 100% and 48.83%, respectively, and the production of 1 kg of syngas required 1.78 kWh of electric energy input. Finally, the obtained results were compared with different plasma methods, including plasma-assisted application coupled with conventional gasification.

## Introduction

Global warming, mostly caused by the anthropogenic impact of the increasing use of fossil fuels for energy production, is currently a vital issue. Therefore, in order to diminish this negative impact, many countries have turned to renewable energy production from available local resources, such as biomass/wastes, hydro, geothermal, wind or sun [1]. Moreover, waste is a permanently and extensively available source generated by each society. Thus, biomass and waste utilization for cleaner and sustainable energy and/or value-added chemicals production contribute to the reduction of greenhouse gases (GHG), carbon footprint and waste streams [2]. Waste-to-energy/fuels concept(s) should also be aligned with a circular economy approach and waste management hierarchy from most to least preferred way of its management [3].

Among all renewable energy sources, biomass (including waste) is distinguished as an alternative organic feedstock to crude oil and natural gas. It has the potential to serve as a backup fuel for combined heat and power production (CHP), thus increasing energy independence. For instance, Lithuania has demonstrated an example of how to become energy independent in a short period of time by using local biomass and waste. During the decade, the share of biomass and waste in the fuel consumption balance shifted from several to more than 70% [4]. However, it is hardly possible that biomass will be capable of fully replacing fossil fuels for the production of chemicals and materials at the current scale and cost [3].

A thermochemical conversion process is one of the most efficient lignocellulosic biomass valorizations to produce energy, biofuels or chemicals. So far, several thermochemical conversion methods have been used, such as incineration, torrefaction, pyrolysis, hydrothermal liquefaction and gasification [5–9]. All these autothermal and allothermal conversion methods enable to effectively convert biomass and waste into gaseous, liquid and solid products. Nevertheless, alternative advanced thermochemical processes have always been investigated in parallel.

Recently, plasma-assisted gasification has received much attention as an emerging technology for circular biomass and waste conversion to recover energy and/or value-added products [10–12]. The use of plasma may overcome limitations specific to conventional waste treatment methods (esterification, anaerobic digestion, incineration, pyrolysis, ‘traditional’ gasification) and enable the recovery of not only energy but also the chemical value of waste [5, 13]. Huang and Tang [6] distinguish two main groups of plasmas: the high-temperature or fusion plasmas and low-temperature plasmas. The low-temperature plasmas may further be divided into thermal plasmas in which a quasi-equilibrium state between electrons and ions is fulfilled and cold plasmas characterized by a non-equilibrium state. The unique properties of thermal plasma, such as high density of energy, high chemical reactivity, very high temperatures (10^3^–10^4^ Kelvin), easy and flexible control, fast start-up/shut-down, high conversion efficiency and lower environmental impact, make plasma promising and attractive method for waste-to-value in the circular economy. However, a highly energy-intensive process, limited process understanding, periodic replacement of wearing parts (e.g. electrodes), and high capital and operational costs prevent this technology from wider commercialization.

Hlina et al. [7] investigated syngas production from biomass and waste using a 100–110 kW power argon/water plasma torch. It was reported that produced syngas featured a very high hydrogen and carbon monoxide content of approx. 90 vol.% with tar concentration under 10 mg/Nm^3^.

Zhang et al. [8] performed gasification of municipal solid waste (MSW) in the Plasma Gasification Melting (PGM) process carrying a 240 kW plasma torch. Air and a mixture of air and steam were used as gasifying agents. It was concluded that the energy efficiency of air/steam gasification of MSW was almost twice higher than that of air gasification, reaching the highest cold gas efficiency (CGE) of approx. 60%.

Shie et al. [9] studied MSW mixed with raw wood gasification using a 10 kW plasma torch. The main reaction component in the producer gas was syngas, which yield increased with the increase of temperature. In contrast, inorganic components were converted into non-leachable and non-hazardous inert slag.

Yoon and Lee [11] carried out microwave (MW) plasma gasification of coal and charcoal. A 5 kW MW plasma generator was used with a mixture of steam and air as a plasma-forming gas. Hydrogen and carbon monoxide content in the syngas ranged between 60 and 75 vol.%. The maximum CGE of approx. 42% was obtained at the gasifying agent/coal ratio of 0.272 (steam 1.1 kg/h and air 20 L/min). It was also determined that the H_2_/CO ratio could be easily adjusted from 3.5 to 0.5 by changing the gasifying agent/coal ratio between 0.0 and 0.544.

Cho et al. [12] used a hybrid gasification system composed of a gasification reactor and a plasma reactor for high-density polyethylene (HDPE) conversion to syngas. A 3 kW direct current (DC) arc plasma torch operating on nitrogen was used to crack unreacted hydrocarbons in the producer gas coming from the solid phase HDPE decomposition in the gasification reactor. The hybrid gasification system achieved a high CGE of 78.8%, similar to a fluidized bed gasifier.

Favas et al. [14] modelled biomass gasification in the plasma environment using the Aspen Plus simulator. Effects of various critical parameters, such as gasification temperature, equivalence ratio (ER) and steam to biomass (SB) ratio on producer gas composition, were carried out. The obtained results indicated that low-temperature plasma gasification was favourable for H_2_ production. High ER had a negative effect on H_2_ production, whereas a high SB ratio positively affected H_2_ production.

Materazzi et al. [15, 16] examined tar and organic sulphur compounds reforming in a two-stage fluid bed–plasma gasification pilot plant using RDF as a feedstock material. The reduction efficiencies exceeded 96%v/v for complex organics (e.g. polycyclic aromatic hydrocarbons (PAH)) and thiophenes. After cleaning with thermal plasma, it was concluded that the syngas was suitable for high-efficiency power production or conversion to biofuels.

Agon et al. [17] investigated plasma gasification of refuse-derived fuels (RDF) using different combinations of gasifying agents (CO_2_ + O_2_, H_2_O, CO_2_ + H_2_O, O_2_ + H_2_O). A 90–160 kW power DC arc plasma torch stabilized with argon/water was used to carry out the experiments. For all studied cases, a medium calorific value syngas with a lower heating value (LHV) up to 10.9 MJ/Nm^3^ was obtained. The carbon conversion efficiency (CCE) ranged from 80 to 100%, and the maximum CGE of 56% was obtained for the steam plasma gasification case. For the latter case, the H_2_/CO ratio was close to 1.95.

Paulino et al. [18] performed the thermodynamic analysis of biomedical waste plasma gasification. The best operating point was defined for produced syngas energy yield of 2.25 at a temperature of 1040 K. The obtained syngas composition was 44.7% H_2_ and 36.98% CO. Authors summarized a general highlight that plasma gasification is a good alternative for processing biomedical waste compared to conventionally applied technologies, such as incineration, autoclaving and microwaves, and gasification.

Chen et al. [19] assessed the performance of a novel medical waste-to-energy design based on plasma gasification and integrated with an MSW incineration plant. The hybrid concept was investigated by multiple approaches, including energy analysis, exergy analysis and economic analysis. It was determined that medical waste-to-electricity’s energy efficiency and exergy efficiency could reach up to 37.83% and 34.91%, respectively. Moreover, the dynamic payback period is only 3.75 years, and the relative net present value is around 45,239.90 k$.

In this experimental research paper, a DC thermal arc plasma torch operating on a mixture of air/water vapor was used for biomass (wood pellets) gasification to syngas. The effects of different gasification parameters, such as the gasifying agent-to-biomass ratio and the power of the plasma torch, on efficient biomass conversion were investigated. The performance of the plasma gasification system based on the main quantification parameters was also assessed and compared.

## Materials and methods

### Feedstock characterization

Wood pellets with the size of 6 mm in diameter were used as a feedstock material for the thermal plasma gasification to syngas. Full proximate and ultimate analyses are described in Table 1.

**Table 1:** Proximate and ultimate analyses of wood pellets

Ultimate (wt.%)		Proximate (wt.%)	
Carbon	51.69±1.1	Volatile matter	78.2±2.84
Hydrogen	6.17±0.02	Fixed carbon	13.62
Nitrogen	<0.01	Ash	0.30±0.01
Sulphur	0.011±0.001	Moisture	7.88±0.84
Oxygen*	42.12	HHV, MJ/kg	19.55±0.41
Chlorine	0.005±0.001	LHV, MJ/kg	18.28±0.45

*By difference

Woody biomass was chosen as well-known reference material to start the experiments with. In the near future, other feedstocks, such as municipal solid waste (MSW), refused-derived fuels and plastics, will be tested in the plasma gasifier.

### Plasma gasifier

**Fig. 1:**
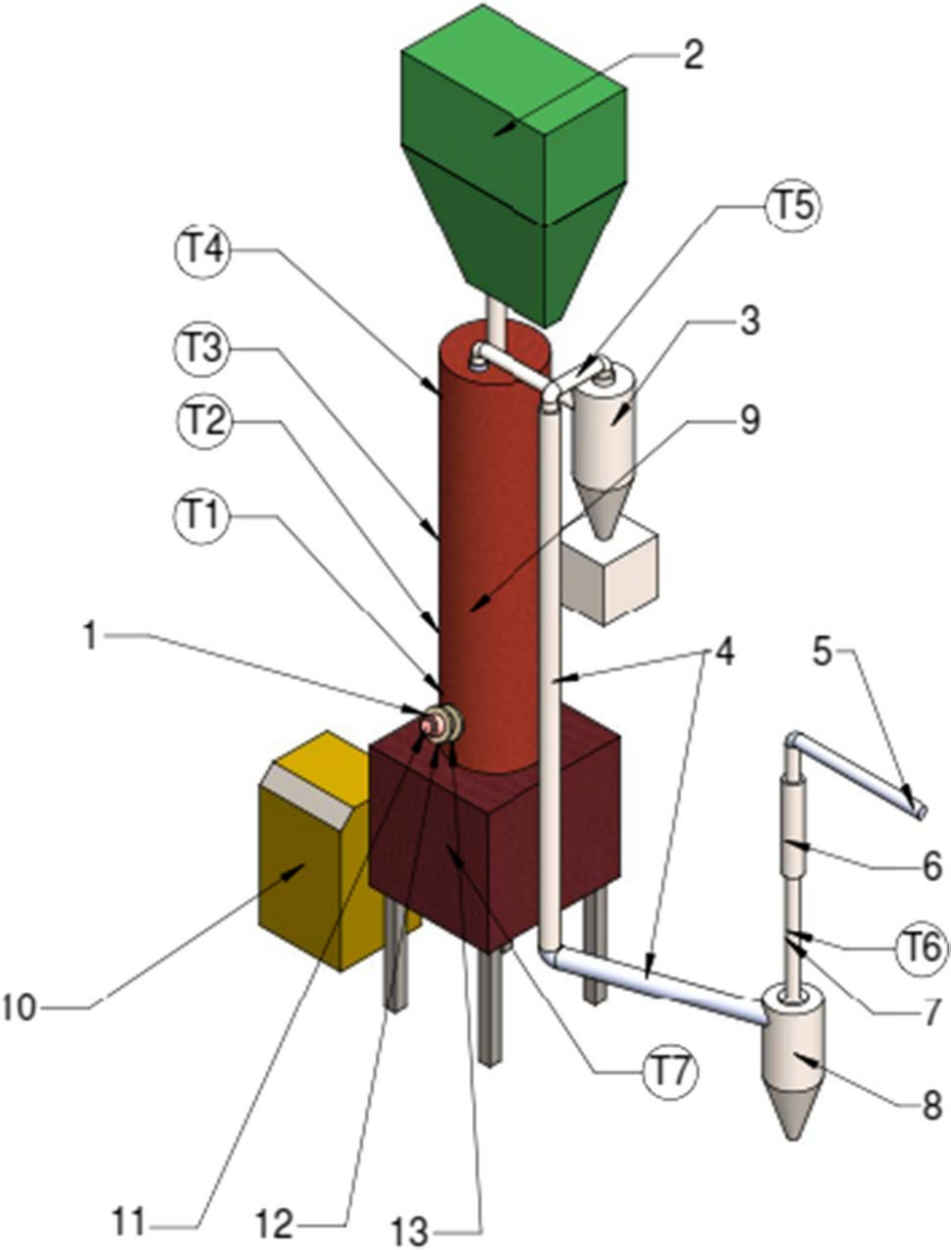
Plasma gasification system

The experimental plasma gasification system was designed at the Plasma Processing Laboratory of the Lithuanian Energy Institute and is shown in Fig. 1.

The main parts of the system consist of the following: 1 – an atmospheric pressure DC arc plasma torch, 2 – a feedstock hopper with a screw feeder, 3 – a cyclone, 4 – a gas cooling (heat exchanger), 5 – a gas burner, 6 – a rotameter, 7 – a gas and tar sampling point, 8 – a condenser, 9 – a plasma-chemical reactor, 10 – an ash-char container, 11 – a power supply, 12 – plasma-forming and shielding gas supply, 13 – a plasma torch cooling, T1, T2, T3, T4, T5, T6, T7 – thermocouples.

The hopper is mounted on top of the plasma-chemical reactor with the biomass feedstock supply through the controlled speed screw feeder. Additionally, 2.0 kg/h of air was used in the hopper to make counter pressure, thus avoiding feedstock clogging. The plasma-chemical reactor has a 25-mm-thick ceramic thermal insulation layer and an inner volume of 0.04 m^3^ (the size is 1.3 m long with a 0.2 m inner diameter). It could be considered a rotating grate updraft gasifier coupled with a plasma torch. The plasma torch is mounted at the bottom of the gasifier. The plasma torch operates on superheated to 240 °C water vapor, which simultaneously serves as a plasma-forming gas, a heat carrier and a gasifying agent. A small constant portion of air of 2.16 kg/h was added as a shielding gas to protect the hafnium cathode from erosion. Depending on the regime, it comprised 10–20% of the total gas flow rate entering the plasma torch.

The gas analyzer SWG 300^−1^ and an Agilent 7890A gas chromatograph equipped with dual-channel thermal conductivity detectors (TDCs) and a valve system were used for gaseous product analysis. The tar content in the producer gas was also measured. The relative error of the obtained results was within limits and was below ±5%. Each experimental point was measured at least three times.

The tar content in the producer gas was measured using a standard method of tar condensation in a solvent (isopropanol, 99.5%), so-called cold trapping. More detailed information on this method is available in [20]. The analysis of tar compounds was performed with a Varian GC-3800 gas chromatograph equipped with a flame ionization detector (FID). Restek RXI-5ms universal 60 m long and 0.25 mm inner diameter capillary column with 0.25-μm-thick (5% phenol) methylpolysiloxane layer was used for chromatographic separation of compounds. Main conditions of measurement: injector temperature – 275 °C, dilution gas ratio 1:75, chromatographic column temperature – from 50 to 325 °C (8 °C/min). Helium, with a 1.2 ml/min flow rate, was used as a carrier gas. The compounds were identified by the characteristic output times obtained by analyzing the calibration mixture EPA 610. Three samples were taken per each experimental point.

### Plasma gasification performance evaluation

Assessing the efficiency of the plasma gasifier, the following gasification performance indicators are usually used such as the producer gas composition (e.g. H_2_, CO, CO_2_, CH_4_, C_x_H_y_), the product gas yield (e.g. H_2_ and CO yield), the H_2_/CO ratio, the LHV of syngas, the CCE, the CGE (or energy conversion efficiency (ECE) by adding plasma power) and the specific energy requirement [21, 22]. Each performance indicator is defined below.

The H_2_/CO ratio indicates the quality of syngas. It is an important parameter showing the potential to produce value-added products from syngas such as chemicals (methanol, methane and hydrogen), synthetic fuels via Fischer–Tropsch (FT) pathway and/or energy (thermal, electrical). Generally, the higher the ratio, the better the syngas quality. However, this depends on the final desired product to be obtained. For instance, the H_2_/CO ratio of 2 is required for Fischer–Tropsch fuels or methanol synthesis, whereas methane synthesis via the Sabatier reaction demands the H_2_/CO ratio of 3 or the H_2_/CO_2_ ratio of 4 [23].

The LHV of syngas:

1 $${\mathrm{LHV}}_{\mathrm{syngas}}=10.78{\mathrm{H}}_2\left(\%\right)+12.63\mathrm{CO}\left(\%\right)+35.88{\mathrm{C}\mathrm{H}}_4\left(\%\right)+56.5{\mathrm{C}}_2{\mathrm{H}}_2\left(\%\right)+64.34{\mathrm{C}}_2{\mathrm{H}}_6+93.21{\mathrm{C}}_3{\mathrm{H}}_8,\left[\frac{\mathrm{MJ}}{\mathrm{N}{\mathrm{m}}^3}\right],$$

where H_2_(%), CO(%), CH_4_(%), C_2_H_2_(%), C_2_H_6_(%) and C_3_H_8_(%) are the content of gaseous products in producer gas.

The H_2_ and CO yield:

2 $$\mathrm{Y}\left({\mathrm{H}}_2\right)=\frac{{\mathrm{m}}_{{\mathrm{H}}_2,\mathrm{OUT}}}{{\mathrm{m}}_{\mathrm{biomass}}}\times 100\%,$$

3 $$\mathrm{Y}\left(\mathrm{CO}\right)=\frac{{\mathrm{m}}_{\mathrm{CO},\mathrm{OUT}}}{{\mathrm{m}}_{\mathrm{biomass}}}\times 100\%,$$

where ${\mathrm{m}}_{{\mathrm{H}}_2,\mathrm{OUT}}$ and m_CO, OUT_ are the mass flow rates of hydrogen and carbon monoxide produced (kg/s), respectively. m_biomass_ is the mass flow rate of biomass feedstock (kg/s).

4 $$\mathrm{Y}\left(\mathrm{gas}\right)=\frac{{\mathrm{V}}_{\mathrm{producer}\ \mathrm{gas}}}{{\mathrm{m}}_{\mathrm{biomass}}},$$

where Y(gas) is the yield of gas produced from 1 kg of feedstock (biomass) (Nm^3^/kg), V_producer gas_ is the volumetric flow rate of the producer gas from the gasifier (Nm^3^/h) and m_biomass_ is the mass flow rate of feedstock (biomass) to the gasifier (kg/h).

The carbon conversion efficiency (CCE):

5 $$\mathrm{C}\mathrm{CE}=12\times {\mathrm{Y}}_{\mathrm{dry}\ \mathrm{gas}}\times \left\{\frac{\left[\mathrm{CO}+{\mathrm{C}\mathrm{O}}_2+{\mathrm{C}\mathrm{H}}_4\right]+2\times \left[{\mathrm{C}}_2{\mathrm{H}}_2+{\mathrm{C}}_2{\mathrm{H}}_6+{\mathrm{C}}_3{\mathrm{H}}_8\right]}{22.4\times \mathrm{C}}\right\}\times 100\%,$$

where Y_dry gas_ is a dry gas yield in Nm^3^ per kg of dry feedstock (Nm^3^/kg), CO, CO_2_, CH_4_, C_2_H_2_, C_2_H_6_ and C_3_H_8_ are in % (v/v), and C is in % of carbon in the dry feedstock.

The energy conversion efficiency (ECE):

6 $$\mathrm{ECE}=\frac{{\mathrm{m}}_{\mathrm{syngas}}\times {\mathrm{LHV}}_{\mathrm{syngas}}}{{\mathrm{m}}_{\mathrm{biomass}}\times {\mathrm{LHV}}_{\mathrm{biomass}}+{\mathrm{P}}_{\mathrm{plasma}}}\times 100\%,$$

where m_syngas_ and m_biomass_ are the mass flow rates of product gas and biomass feedstock (kg/s), respectively. LHV_syngas_ and LHV_biomass_ are net calorific values of product gas and biomass feedstock (MJ/kg), respectively. P_plasma_ is a plasma torch power (kW).

The specific energy requirements (SER):

7 $$\mathrm{SER}=\frac{{\mathrm{P}}_{\mathrm{plasma}}}{{\mathrm{m}}_{\mathrm{syngas}}},$$

where SER is the specific energy requirement to produce 1 mol or kg of syngas (kJ/mol or kWh/kg), P_plasma_ is the plasma torch power (kJ/s) and m_syngas_ is the mass flow rate of syngas gas (mol/s).

## Results and discussion

### Effect of the water vapor-to-biomass ratio on gasification performance

The effect of the water vapor-to-biomass ratio (WB) on biomass gasification efficiency was studied in this section. The mass flow rate of water vapor was in the range of 8.64–16.74 kg/h (+ 2.16 kg/h of air used as a cathode shielding gas), while the feeding rate of wood pellets through the screw feeder was kept constant at 19.44 kg/h. This gave the water vapor-to-biomass ratio of 0.56–0.97. At these conditions, the power of the plasma torch varied from 43.7 to 71 kW (arc current of 180–200 A, arc voltage of 240–355 V, plasma torch thermal efficiency of 0.433–0.543).

**Fig. 2:**
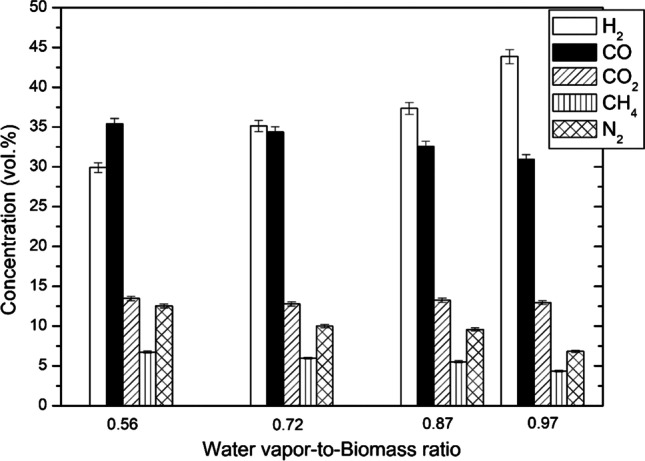
Elemental composition of the producer gas

Figure 2 shows the elemental composition of the producer gas after biomass gasification. The main gaseous reaction products were hydrogen and carbon monoxide, both comprising more than 65–75% of the total gas produced. The remaining part was carbon dioxide, methane and nitrogen. Some traces of C_2_H_2_ (1.0–1.6%), C_2_H_6_ (0.05–0.3%) and C_3_H_8_ (0.01–0.08%) were also obtained. NO_x_ (8–30 ppm) and SO_2_ (1144–1425 ppm) were also present due to air used as a shielding gas and a counterpressure gas in the hopper and sulphur present in the biomass. As the WB ratio increased from 0.56 to 0.97, the concentration of H_2_ increased, and CO decreased because of the dominance of steam reforming and water–gas shift (WGS, CO + H_2_O ↔ H_2_ + CO_2_) reactions.

**Fig. 3:**
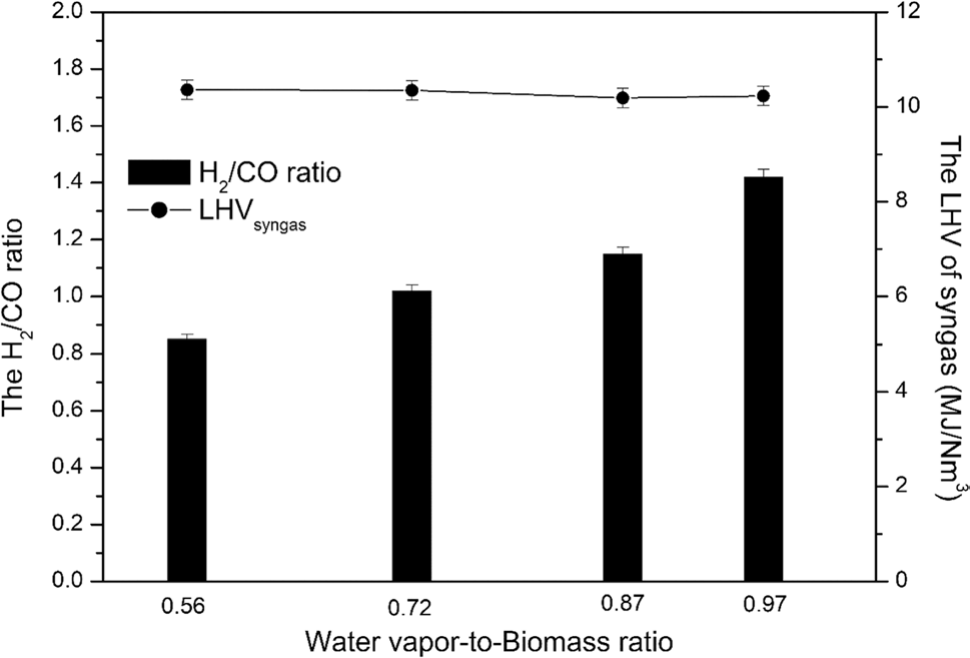
Effect of the WB ratio on the H_2_/CO ratio and the LHV of the syngas

As the WB ratio increased, the H_2_/CO ratio increased from 0.85 to 1.42, while the LHV of the syngas did not change much and was in the range of 10.2–10.36 MJ/Nm^3^ (Fig. 3). The H_2_/CO ratio indicates that the produced syngas is not suitable for direct synthetic fuels production via Fischer–Tropsch synthesis and therefore the proper ratio adjustment is needed via WGS reaction.

**Fig. 4:**
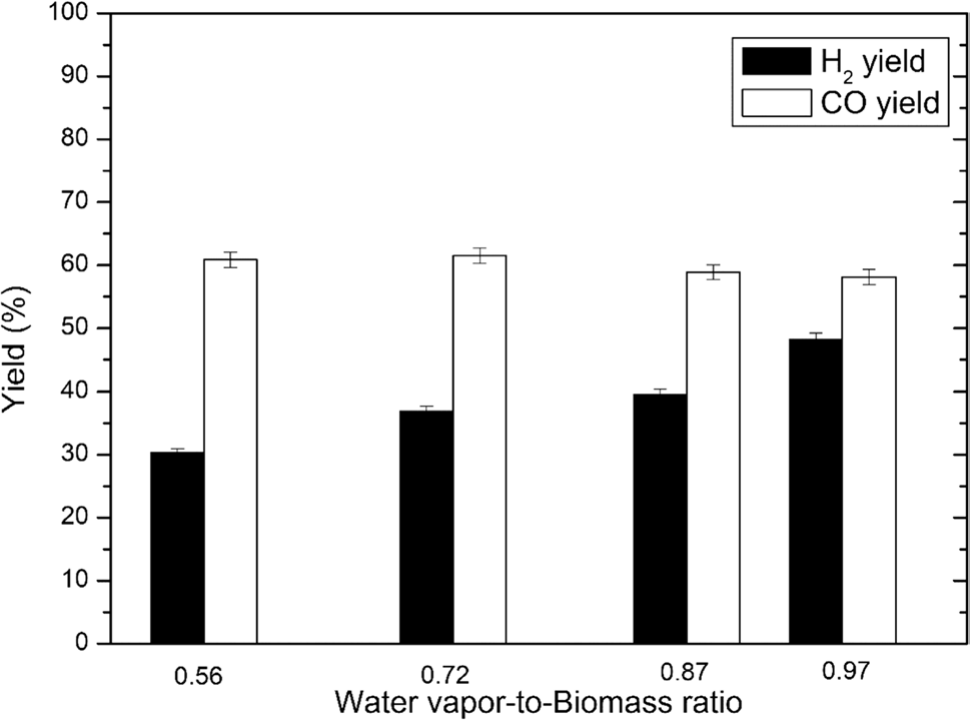
Effect of the WB ratio on the yield of H_2_ and CO

The effect of the WB ratio on the yield of H_2_ and CO is shown in Fig. 4. It could be seen that as the WB ratio increased from 0.56 to 0.97, the yield of H_2_ increased from 30.3 to 48.3%, whereas the yield of CO remained within the limits of 58.13–61.54%, thus having a tendency to decrease at the higher WB ratio slightly. Hydrogen yield was mostly affected by increasing the water vapor flow rate from 8.64 to 16.74 kg/h during steam reforming with biomass reaction. Additionally, part of H_2_ came from biomass conversion as well. As the biomass feeding rate was constant, the yield of CO did not change much with a major part of carbon and oxygen coming from the gasification of wood pellets (Table 1).

**Fig. 5:**
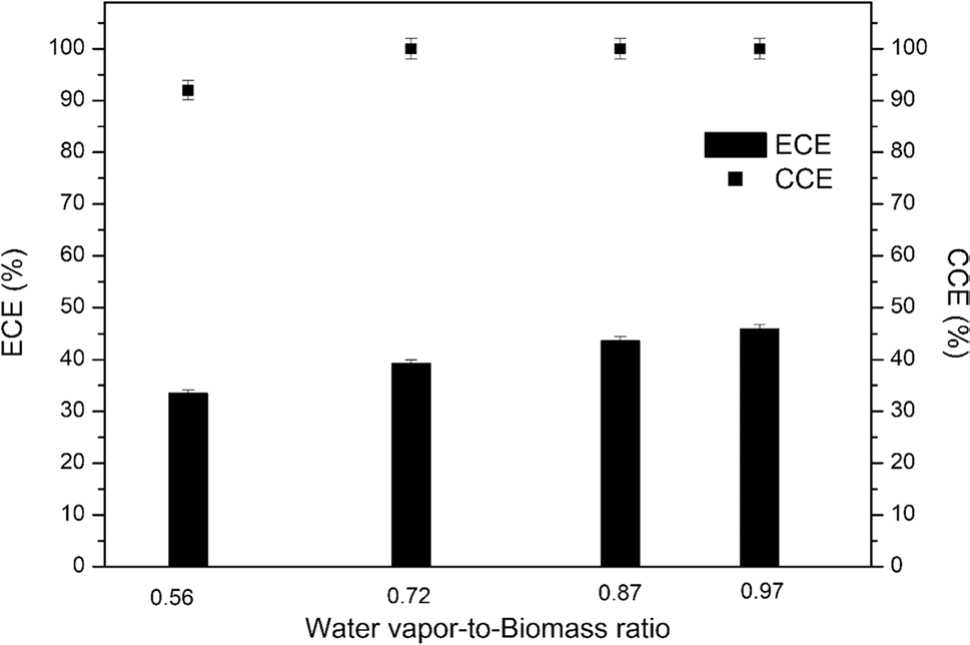
Effect of the WB ratio on the ECE and CCE

The effect of the WB ratio on the ECE and CCE is shown in Fig. 5. As the WB ratio increased, both the ECE and the CCE increased. The highest value of 45.8% of ECE was achieved at the WB ratio of 0.97. In this experimental regime, the gasification efficiency was optimum even if the power of the plasma torch was the highest at 71 kW. At the WB ratio of 0.56, 0.72 and 0.87, the plasma torch power was 43.7 kW, 54.9 kW and 57.1 kW, respectively. Thus, the higher yield of produced syngas compensates for the increased power consumption by the plasma torch. The CCE reached 100% at the WB ratio of 0.72 and remained the same at 0.97.

**Fig. 6:**
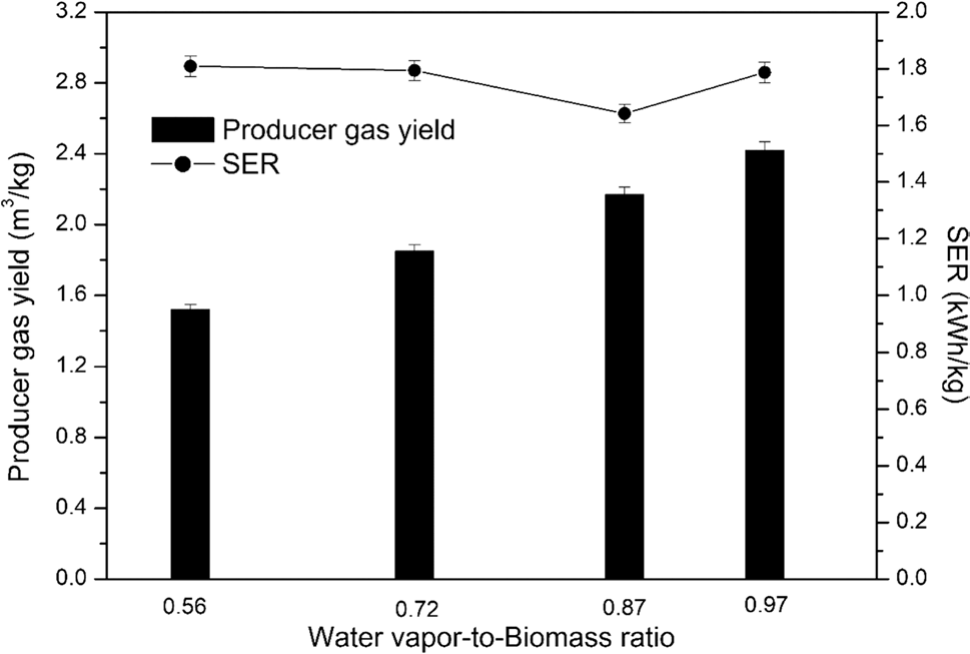
Effect of the WB ratio on the producer gas yield and the SER

Figure 6 shows the dependence of the WB ratio on the producer gas yield and the SER. As the WB ratio increased from 0.56 to 0.97, the producer gas yield increased from 1.52 to 2.42 Nm^3^/kg biomass. This was mostly attributed to the increased water vapor flow rate simultaneously serving as a plasma-forming gas, a heat carrier and a gasifying agent. The SER required to produce 1 kg of syngas from wood pellets had a tendency to decrease from 1.81 to 1.642 kWh/kg at the WB ratio in the range of 0.56–0.87. However, the SER increased from 1.642 to 1.787 kWh/kg at the WB ratio of 0.97. It was due to increased arc current from 180 to 200 A of the plasma torch because its operation at 180 A and water vapor flow rate at 16.74 kg/h was unstable. Therefore, to ensure a stable operation, the current was increased, which directly affected the power of the plasma. As a result, due to increased power, the SER increased. Even the increased production of syngas could not compensate for increased energy demand.

**Fig. 7:**
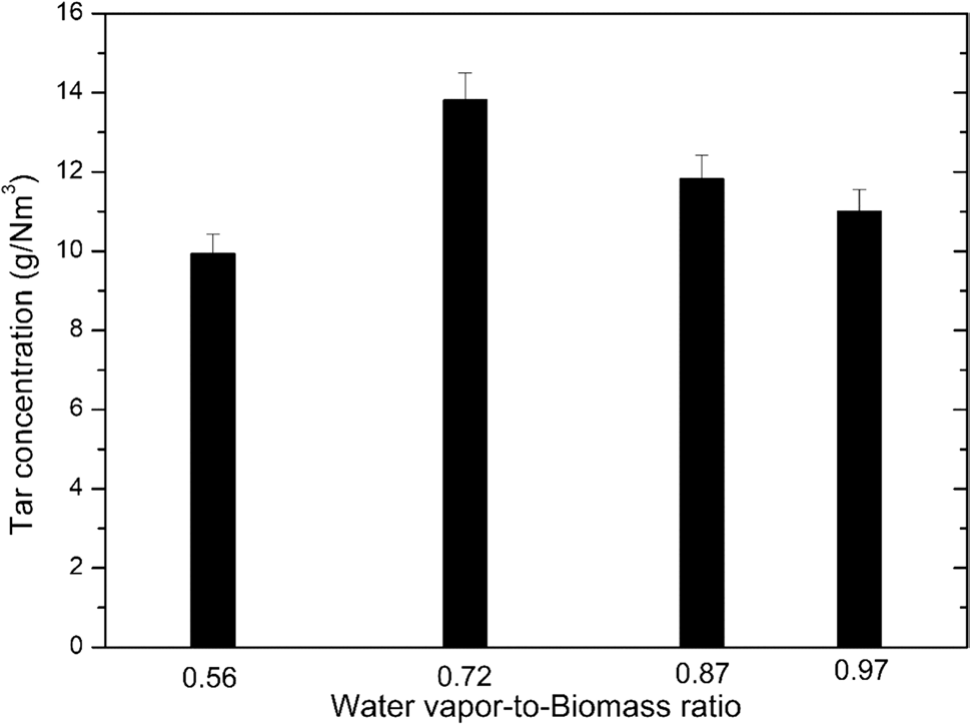
Effect of the WB ratio on the tar content in the producer gas

The measured tar content in the producer gas is shown in Fig. 7. As the WB ratio increased from 0.56 to 0.97, the concentration of tars with some fluctuations increased from around 9.937 to 11.0 g/Nm^3^, respectively. However, the highest concentration of 13.81 g/Nm^3^ was detected at the WB ratio of 0.72. The increasing tar content could be explained by a shorter residence time inside the plasma-chemical reactor due to the increasing flow rate of water vapor. Generally, experimentally obtained high tar content is not typical for the plasma conversion method. The tar content reported by Hlina et al. [7] converting biomass (pellets, sawdust) and waste to syngas was below 10 mg/Nm^3^. Moreover, wood pellets’ energy density is higher than wood chips, and a higher residence time is needed to crack tar compounds fully. The major tar constituents were benzene and toluene, which comprised more than 40–60% of the total mixture. The possible solution for reducing tars is either to increase the residence time by enlarging reactor size, which is rather complicated, or reducing the flow rate of feeding material or increasing the power of the plasma torch, especially the arc current. The latter is easily possible; however, increased energy consumption could decrease overall energy efficiency.

**Fig. 8:**
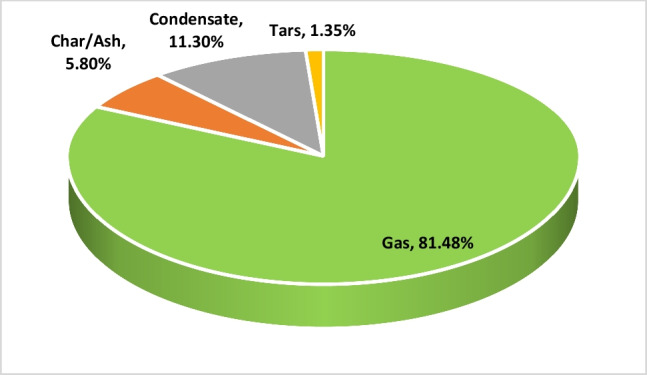
Reaction products composition after the thermal plasma biomass gasification

The effect of the water vapor-to-biomass ratio on mass and energy flows is described in this section. The results are summarized at the best experimental process conditions, i.e. WB ratio of 0.97. Reaction products composition after wood pellets gasification in a thermal plasma environment is shown in Fig. 8. It could be seen that the dominant reaction product is gas, or syngas, constituting more than 81.48% of the total mass of the products, followed by condensate (11.30%), char/ash (5.80%) and tars (1.35%). As mentioned above, the tar content is too high; however, it could be reduced by improving reactor design or adjusting experimental parameters to increase residence time. After the experiments, the collected char/ash was characterized by performing the proximate and ultimate analysis (Table 2). Despite the significant reduction of the volatile fraction of the raw material (Table 1), the obtained solid residual part still has some volatiles. However, the major dominant part is carbon/fixed carbon with a small amount of ashes. Moreover, the energy content of the char is relatively high and could be reused back in the process by mixing with wood pellets. Generally, the solid part could be reduced to 1–2% as thermal plasma allows it to do so due to very high temperatures.

**Table 2:** Proximate and ultimate analysis of residual char/ash

Ultimate (wt.%)		Proximate (wt.%)	
Carbon	89.60±0.79	Volatile matter	11.56±0.08
Hydrogen	1.95±0.28	Fixed carbon	84.08
Nitrogen	0.15±0.01	Ash	2.69±0.08
Sulphur	0.005±0.001	Moisture	1.67±0.02
Oxygen*	8.28	HHV, MJ/kg	32.14±0.13
Chlorine	0.013±0.003	LHV, MJ/kg	31.72±0.13

*By difference

**Fig. 9:**
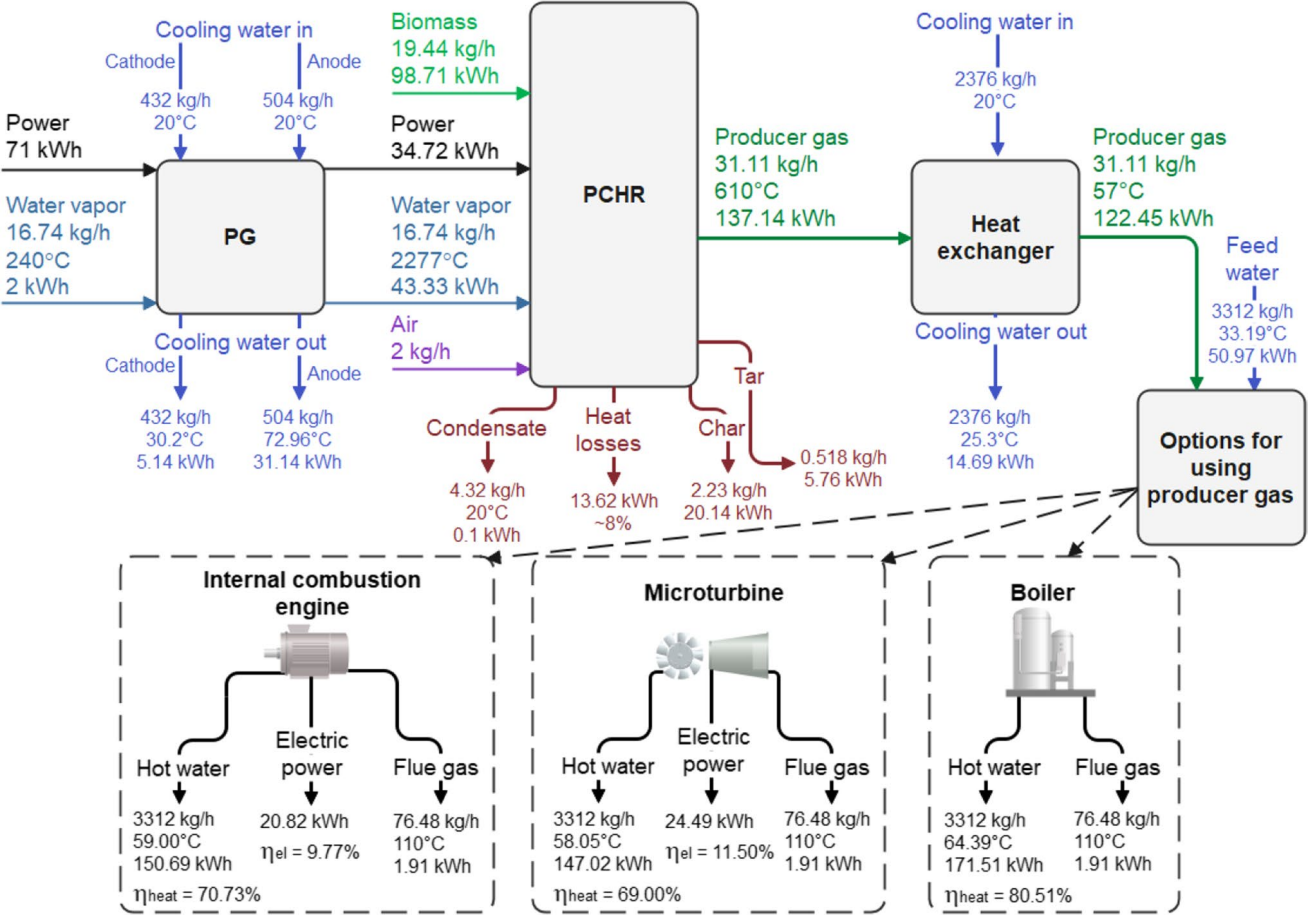
Energy and mass flow of thermal plasma gasification of wood pellets at the WB ratio of 0.97. PG, plasma generator; PCHR, plasma-chemical reactor

The energy and mass balance of wood pellets gasification in the thermal arc water vapor plasma is presented in Fig. 9. At the WB ratio of 0.97, 71 kWh of electrical energy was needed to run the plasma torch to generate the water vapor plasma jet, which served as a main gasifying agent and heat carrier. However, only half of the electrical energy (34.72 kWh) was transferred to useful heat through the Joule heating to the chemical reactor by the plasma jet, while the remaining part of 36.28 kWh (~51.1%) was lost to water cooling the plasma torch. Therefore, the thermal efficiency of the plasma torch, depending on the experimental conditions, was in the range of 0.43 to 0.54. Typically, the thermal efficiency of 0.4–0.9 could be obtained [24]. This depends on the construction design of the plasma torch, plasma type (DC arc, MW, RF, etc.) and plasma-forming gas used. Striugas et al. [25] reported a 0.81 plasma torch thermal efficiency investigating sewage sludge treatment using air plasma–assisted gasification. For steam and liquid plasma torches, the thermal efficiency could be in the range of 0.4–0.75 [26, 27]. The (updraft) plasma gasifier used 19.44 kg/h of wood pellets to produce hot gas containing 137.14 kWh of energy. An extra 43.33 kWh of energy came from the sensible heat of water vapor, which was heated to 2277 °C by the electric arc inside the discharge chamber of the plasma torch. Therefore, the energy conversion efficiency, the parameter similar to the hot/cold gas efficiency of the conventional gasifier, calculated from Eq. 6, was 45.83% (Fig. 5). Also, part of the energy in the gasifier was lost to char/ash and tars. These losses comprised 11.4% (or 20.14 kWh) and 3.25% (or 5.76 kWh), respectively. Moreover, around 8% (or 13.62 kWh) of energy was lost due to radiation, while the energy losses to condensate were negligible. Downstream of the plasma gasifier, the producer gas was cooled down to 57 °C, and 14.69 kWh of heat was transferred to the water. The producer gas cooling step in the heat exchanger could be avoided if it is directly burnt in a boiler for heat production. However, this step was mostly needed while calculating the energy balance more accurately. Despite this, three options utilizing producer gas could be proposed. Besides the first option mentioned above (calculated according to Eq. 8), the other is to use electrical power generation devices, such as an internal combustion engine (ICE) and/or a microturbine (MT). Considering the conversion performance efficiency of the producer gas into electricity by the ICE (17%) and the MT (20%) [21, 28], the calculated electrical efficiency (Eq. 9) could be 9.77% and 11.50%, respectively. This is comparable to the efficiency of 11.7% of the electricity production of the CHP process using a 75 kW_el_ Stirling engine (the efficiency of the engine is 26.8%) [22]. Karellas et al. [29] reported approx. 12–18% total electrical efficiency coupling allothermal biomass gasification with a microturbine for CHP production. The electrical efficiency of the turbine (Capstone C30) used in the study was 26%. Additionally, syngas utilization for higher added value products, such as methane, methanol or hydrogen, production could be an option. However, this case was not considered in this research.

Thermal efficiency of the syngas utilization:

8 $${\upeta}_{\mathrm{heat}}=\frac{{\mathrm{Q}}_{\mathrm{hw}}}{{\mathrm{Q}}_{\mathrm{biomass}}+{\mathrm{Q}}_{\mathrm{Wv}}+{\mathrm{P}}_{\mathrm{plasma}}}\times 100\%,$$

where Q_hw_ is an energy content accumulated in hot water (kWh), Q_biomass_ is an energy content in wood pellets (kWh), Q_wv_ is an energy content (sensible heat) in water vapor as a plasma-forming gas (kWh) and P_plasma_ is the plasma torch power (kWh).

Electrical efficiency of the syngas utilization:

9 $${\upeta}_{\mathrm{el}}=\frac{{\mathrm{P}}_{\mathrm{el}}^{\mathrm{out}}}{{\mathrm{Q}}_{\mathrm{biomass}}+{\mathrm{Q}}_{\mathrm{Wv}}+{\mathrm{P}}_{\mathrm{plasma}}}\times 100\%,$$

where ${\mathrm{P}}_{\mathrm{el}}^{\mathrm{out}}$ is an electrical energy content in the internal combustion engine or microturbine gained from producer gas (kWh).

### Comparison between results

In this section, the experimental results gasifying various types of biomass and waste in the ambient of thermal plasma are summarized. The results are shown in Table 3.

**Table 3:** Summary of biomass and waste gasification to syngas in thermal plasma

Feedstock	P_plasma_,kW	Gasifying agent	H_2_,vol.%	CO, vol.%	H_2_**/**CO ratio	LHV_syngas_,MJ/Nm^3^	CCE, %	ECE,%	SER, kWh/kg	Ref.
Sawdust	DC arc, 110	CO_2_	41.6	50.9	0.81	-	-	-	-	[7]
Pellets		CO_2_	43.8	51.7	0.85	-	-	-	-	
Plastics		CO_2_	41.6	49.7	0.84	-	-	-	-	
Oil		H_2_O	59.5	30.2	1.97	-	-	-	-	
RDF	DC arc, 90–160	CO_2_ + O_2_	30	46	0.65	9.9	86	48	-	[17]
		H_2_O	52.7	27	1.95	10.7	84	56	-	
		CO_2_ + H_2_O	37	42	0.88	10.5	82	54	-	
		O_2_ + H_2_O	45	37	1.22	10.4	100	53	-	
MSW	DC arc, 240	Air	20	13	1.5	6.0	-	44	-	[8]
		Air + H_2_O	19	10.5	1.8	7.0	-	53	-	
HDPE^a^	DC arc, 3	N_2_+O_2_+H_2_O	25.7	35.9	0.71	-	-	78.8	-	[12]
MSW/RW^b^	DC arc, 10	N_2_ + H_2_O	53.34	36.51	1.46	-	-	-	-	[9]
MSW^c^	DC arc, 400	Air	10.4	14.2	0.73	-	-	-	1.14^f^	[30]
Coal	DC arc, 5	Air + H_2_O	45	29	1.55	-	52	43	-	[11]
		Air	32	43	0.74	-	62	37	-	
		H_2_O	62	22	2.8	-	28	30	-	
Charcoal		Air + H_2_O	48	23	1.8	-	26	24	-	
		Air	22	43	0.51	-	32	18	-	
		H_2_O	58	17	3.4	-	18	25	-	
Waste cooking oil	DC arc, 57.6	H_2_O^d^	47.9	22.42	2.14	12.7	66.1	54.29	1.8	[31]
Glycerol	DC arc, 62.4	H_2_O^d^	51.16	24.74	2.07	9.82	100	63.86	1.77	[32]
	DC arc, 56	Air	29	27	1.07	7.32	75.7	43.64	2.47	
Glycerol	DC arc, 57	pure H_2_O^e^	57.9	21	2.76	10.16	68	39.5	2.11	[33]
Glycerol	MW, 2	Air + H_2_O	57	35	1.63	12	100	-	-	[34]
Glycerol	DC arc, 24.1	Ar + water	56	38	1.47	11	100	66	-	[35]
Olive pomace charcoal	DC arc, 52.2	H_2_O^d^	41.17	13.06	3.15	6.09	-	-	-	[36]
Wood pellets	DC arc, 71	H_2_O^d^	43.86	30.93	1.42	10.23	100	45.83	1.78	This work

^a^*HDPE*, high-density polyethylene, hybrid gasification-plasma system. Plasma is used for gas reforming and cracking

^b^*RW*, raw wood

^c^Integrated gasification-vitrification pilot plant for direct MSW treatment. Plasma is used for slag melting and gas cleaning

^d^Additionally, the air was used as a cathode shielding gas comprising 10–20% of the total flow rate of the plasma-forming gas

^e^Pure water vapor is used as a plasma-forming gas with no admixture of other cathode protective gases

^f^Electricity needed per 1 kg of MSW treated, not the electricity required to produce 1 kg of syngas according to Eq. 7

As could be seen from the above table, there is a number of research dedicated to various kinds of biomass and waste conversion to syngas. The DC arcs both transferred and non-transferred are the dominant sources for plasma generation, with the latter being more prevalent. This dominance of the DC arc plasma torches could be related to higher technological robustness, lower complexity and a relatively cheaper method compared to microwave plasma. Moreover, the use of DC arc enables the operation of a wide range of plasma powers, starting from several kilowatts up to hundreds of kilowatts or even megawatts [37]. Despite this, the plasma method currently has limited industrial application in a circular economy due to higher capital (CAPEX) and operational (OPEX) expenditures [38].

Various gasifying agents have been investigated, which is a very important parameter producing higher quality syngas. According to the summarized results, utilization of water vapor, both pure or in a mixture with other gases, yields a higher H_2_ concentration in the producer gas, thus enabling to obtain higher H_2_/CO ratio and LHV. The use of air is less efficient due to nitrogen and NOx compounds present in the producer gas. As a result, the gasification performance efficiency using air is lower. Contrary to the technological perspectives, the use of water vapor instead of air as a gasifying agent and a plasma-forming gas is a challenging issue. From previous personal experimental investigations, it was determined that the lifetime of the electrodes of the plasma torch operating on water vapor is shorter due to its condensation on the discharge chamber walls. Therefore, various shielding gas, such as N_2_, Ar and Air, are being used.

The energy conversion efficiency (equivalent parameter in ‘traditional’ gasification is a cold gas efficiency) reported in this table varies from around 18 to 60%. The exception is 78.8% reported in [12]. Generally, the average ECE value using water vapor as a gasifying agent is around 50–55%, which is still lower compared to conventional gasification, such as a fluidized bed with a CGE of ~80% [39]. The use of air even lowers the ECE to around 18–43% due to ballast nitrogen. The lower performance efficiency of thermal plasma gasification in terms of the ECE could be compensated by combining conventional gasification with plasma-assisted producer gas cleaning. For instance, using plasma for tar cracking and gas reforming, thus avoiding expensive producer gas conditioning [22, 26, 27].

The energy required to produce 1 kg of syngas or the energy per kilogram of treated feedstock is only reported by several researchers, Tamošiūnas et al. [31–33] and Byun et al. [30], respectively. Byun et al. [30] reported the SER of 1.14 kWh per kilogram of MSW treated in the integrated demonstration gasification/vitrification unit for MSW, with a 10 tons/day capacity. Tamošiūnas et al. [31–33] indicated the SER per kilogram of syngas produced in the range of 1.77 to 2.47 kWh gasifying different types of biomass and waste, including this research. However, the research was performed with a lab-scale plasma gasifier.

Generally, it could be stated that plasma gasification is a promising and efficient method for biomass and waste valorization to value-added products. Alternatively, in order to increase the conversion process performance, thermal plasma could be coupled with conventional gasification technologies. For instance, the above-mentioned thermal plasma tar reforming and gas upgrading or additional processing of ash/char to vitrified slag remaining after traditional gasification. Further research needs to be carried out to get more data about a wider range of plasma gasification performance parameters and process optimization.

## Conclusions

In this experimental study, wood pellets’ gasification to syngas was investigated by DC thermal arc plasma at atmospheric pressure. Water vapor was used as a main gasifying agent, a plasma-forming gas and a heat carrier. The plasma gasification system was quantified in terms of the producer gas composition, the tar content, the H_2_/CO ratio, the carbon conversion efficiency, the energy conversion efficiency and the specific energy requirements. It was determined that the biomass gasification performance efficiency was highest at the water vapor-to-biomass ratio of 0.97, i.e. plasma-forming gas flow rate of 18.74 kg/h (16.74 kg/h of water vapor and 2.16 kg/h of air), the biomass flow rate of 19.44 kg/h and plasma torch power of 71 kW). The producer gas was mostly composed of H_2_ (43.86 vol.%) and CO (30.93 vol.%), giving the H_2_/CO ratio of 1.42 and the LHV of 10.23 MJ/Nm^3^. The tar content obtained in the syngas was in the range of 9.937 to 13.81 g/Nm^3^. The yields of H_2_ and CO were 48.31% and 58.13%, respectively, with the highest yield of the producer gas of 2.42 Nm^3^/kg biomass. The carbon conversion efficiency and the energy conversion efficiency were 100% and 48.83%, respectively, and the production of 1 kg of syngas required 1.78 kWh of electric energy input. Moreover, the mass and energy balances of the plasma gasification process were defined at the WB ratio of 0.97 and options for utilizing syngas were proposed. Finally, the obtained results were compared with different plasma methods, including plasma-assisted application coupled with conventional gasification. Further experiments are planned, converting used COVID-19 medical masks to syngas.

## Data Availability

Not applicable.
